# How Does Socioeconomic Development Affect COPD Mortality? An Age-Period-Cohort Analysis from a Recently Transitioned Population in China

**DOI:** 10.1371/journal.pone.0024348

**Published:** 2011-09-15

**Authors:** Jing Chen, Catherine Mary Schooling, Janice Mary Johnston, Anthony Johnson Hedley, Sarah Morag McGhee

**Affiliations:** Department of Community Medicine and School of Public Health, The University of Hong Kong, Hong Kong; Statens Serum Institute, Denmark

## Abstract

**Background:**

Chronic obstructive pulmonary disease (COPD) is a leading cause of death, particularly in developing countries. Little is known about the effects of economic development on COPD mortality, although economic development may potentially have positive and negative influences over the life course on COPD. We took advantage of a unique population whose rapid and recent economic development is marked by changes at clearly delineated and identifiable time points, and where few women smoke, to examine the effect of macro-level events on COPD mortality.

**Methods:**

We used Poisson regression to decompose sex-specific COPD mortality rates in Hong Kong from 1981 to 2005 into the effects of age, period and cohort.

**Results:**

COPD mortality declined strongly over generations for people born from the early to mid 20th century, which was particularly evident for the first generation to grow up in a more economically developed environment for both sexes. Population wide COPD mortality decreased when air quality improved and increased with increasing air pollution. COPD mortality increased with age, particularly after menopause among women.

**Conclusions:**

Economic development may reduce vulnerability to COPD by reducing long-lasting insults to the respiratory system, such as infections, poor nutrition and indoor air pollution. However, some of these gains may be offset if economic development results in increasing air pollution or increasing smoking.

## Introduction

With economic and social development as well as the epidemiologic transition, chronic noncommunicable diseases are projected to become the leading cause of deaths in both developed and developing countries [1]. Chronic obstructive pulmonary disease (COPD) is one of the identified chronic noncommunicable diseases among the leading causes of death which will pose a huge burden to society. COPD is characterized by airflow limitation which is usually caused by an abnormal inflammatory response of the lungs to noxious particles or gases and is not fully reversible [2]. It is a preventable and treatable disease, with the environmental drivers including smoking, secondhand smoking, poor air quality throughout life, occupational exposures and childhood infections [3]. According to WHO estimates, 210 million people have COPD and 3 million people died from COPD in 2005 corresponding to 5% of all deaths globally. Meanwhile, COPD is predicted to be the third leading cause of death worldwide by 2030 [4], and usually entails years of morbidity before death with reduced quality of life and high health care costs.

Despite the heavy burden of COPD, there are few analyses of the impact of economic development on the trend in mortality due to COPD [5]. However, in developing countries, a more rapid epidemiologic transition is taking place than has previously occurred in developed countries [6], which provides an opportunity to examine any possible different impacts on mortality. Uniquely in Asia, the Hong Kong population has already experienced rapid and recent economic development over a lifetime, that is, in the last seventy years. This development has been marked by changes at clearly identifiable time points. For example, the Hong Kong population was largely formed by mass migration from pre-industrial China to comparatively developed Hong Kong in the mid-20^th^ century [7]. In Hong Kong itself there was rapid economic growth from pre-industrial to post-industrial levels over the subsequent forty years, with corresponding implications for air pollution, albeit temporarily mitigated by a control on the sulfur content in fuel in 1990 [8]. This unique setting provides a rare opportunity to investigate the impact of socio-economic development and macro-level interventions on trends in COPD mortality. In common with other recently developed populations, there is little information on trends in the prevalence of smoking. However, in Hong Kong, the prevalence of smoking among women is low compared to that among men [9], allowing a study of the effects of economic development almost independent of active smoking among women.

We used an age period cohort (APC) model to decompose sex-specific COPD mortality into the effects of age, period and cohort, in order to examine the contributions of economic development and associated exposures. These include the contribution of population wide reductions in air pollution, particularly ambient sulphur dioxide in 1990s [8] which would be expected to be seen as a period effect in the APC model and differences between generations, such as the change in childhood living conditions in the mid 20^th^ century, which would be expected to be seen as a cohort effect in the APC model.

## Methods

### Sources of Data

Annual deaths from COPD (International Classification of Diseases (ICD)-9 codes 490–496 and ICD-10 codes J40-47) by age and sex from 1981 to 2005 were abstracted from the death registration data of the Department of Health, Hong Kong SAR Government [10]. We obtained the age and sex specific mid-year populations of Hong Kong for the same years from the Census and Statistics Department [11]. Deaths from COPD in people below 45 years old were rare; the total number of deaths was 897 for both sexes from 1981 to 2005 [10]. Therefore only those aged 45 and above were included in this analysis. The ICD-9 and ICD-10 coding systems for mortality from chronic lower respiratory diseases are comparable [12], hence deaths for 1981–2005 were considered together.

### Statistical analysis

Deaths from COPD were grouped into nine 5-year age groups, from 45–49 years to 85+ years. Time period of death was grouped into five 5-year intervals from 1981–85 to 2001–05. Birth cohorts were constructed by subtracting age of death from period of death (cohort = period-age). This gave a total of 13 synthetic birth cohorts since 1896 centered at 5-year intervals. Men and women were considered separately. A log-linear regression model was fitted to the death data using maximum likelihood in order to estimate the effects of age, period and cohort by assuming a Poisson distribution for the observed deaths from COPD [13]. Thus, given d*_ij_* is the observed number of deaths for age group *i* in time period *j*, then the mean μ*_ij_* of d*_ij_* is assumed to follow a Poisson distribution (*d_ij_∼Poisson (μ_ij_)*) and:
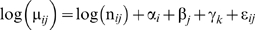
where α*_i_* is the age effect (*i* = 1, …I), β*_j_* is the period effect (*j* = 1, …J) and γ*_k_* is the cohort effect (*k* = 1, …K), where I, J and K stand for age groups, calendar periods and birth cohorts respectively. ε*_ij_* is the random error term and n*_ij_* denotes the total number of person years.

In age-period-cohort (APC) models it is impossible to identify the role of age, period and cohort separately in the full model, since these three variables are not independent from each other. To overcome this problem, we adopted the commonly used solution of constraining the period effect to have two reference points [14], which were the periods 1986–1990 and 1996–2000. We also used the 70–74 year age group and the 1936–1940 birth cohort as reference points. Use of different references points did not materially alter the results. The full APC model was compared with three partial models including age only, age and cohort (AC) as well as age and period (AP). The deviance of the model was used to measure the goodness of fit of each model. The statistical significance of the difference in deviances between the subsequent models was assessed by a log-likelihood ratio test. A smaller deviance value indicates a better model fit to the data. The estimations were done using SAS version 9.1.

## Results

**Figure 1:**
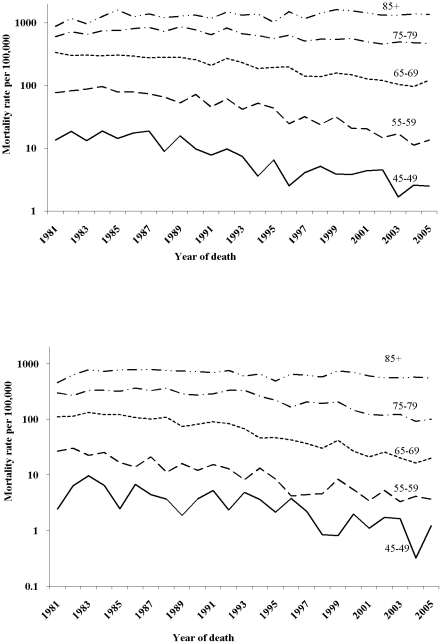
Age specific mortality rates from COPD in men (above) and women (below) by year of death in Hong Kong, 1981–2005.

**Figure 2:**
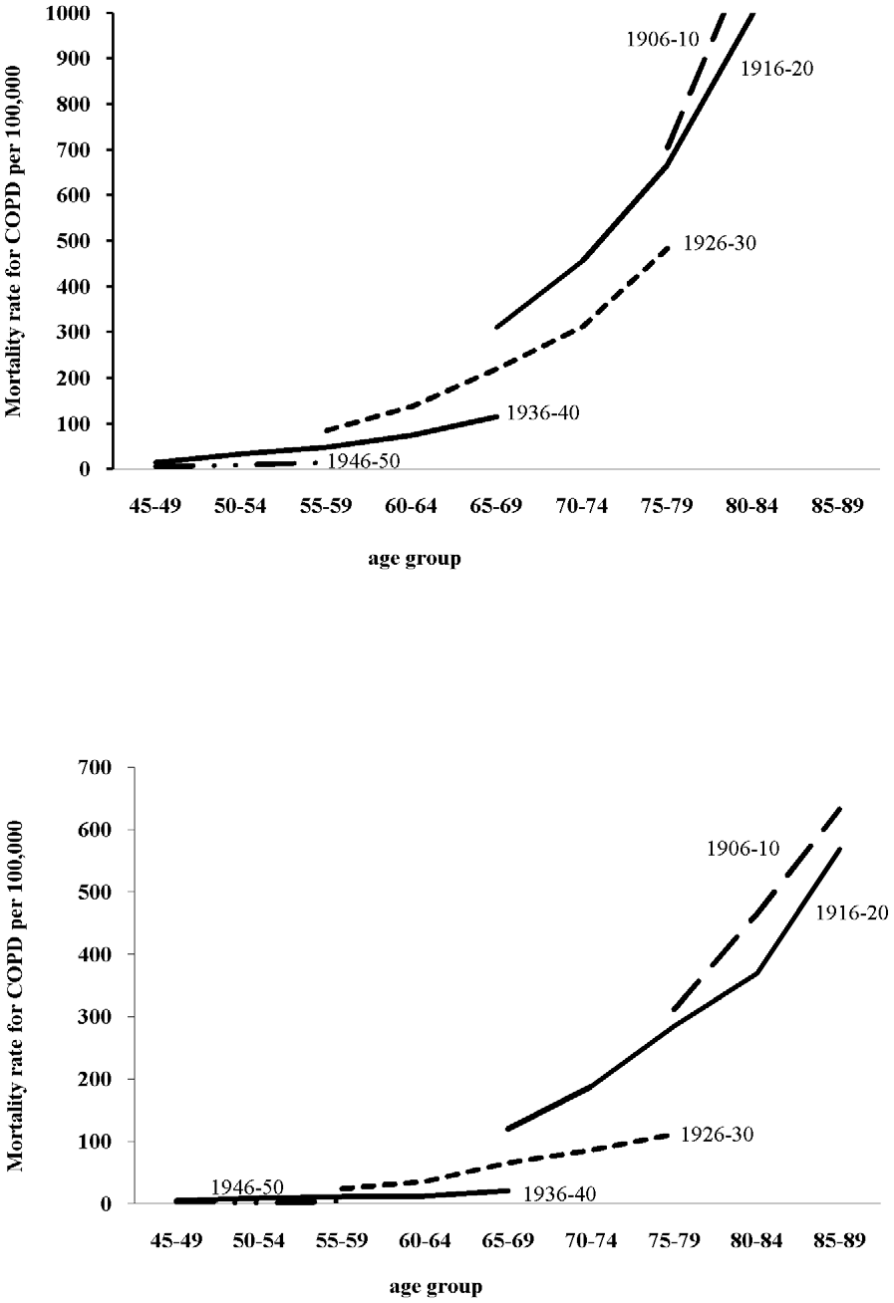
Age specific mortality rates from COPD men (above) and women (below) by birth cohorts in Hong Kong.

As expected, when looking at age specific mortality rates on a logarithmic scale, they were much higher in older people for men and women and declined throughout the period except in the oldest age group, where they increased slightly (Figure 1). As to the age specific mortality rates for every other 5-year birth cohort, the curves show decreasing mortality with each successive generation for men and women (Figure 2). The change in deviance (i.e. goodness of fit) in the sequence of model building showed that both age-period (AP) and age-cohort (AC) models significantly improved the fitness over the age only model. The full APC model in turn was better than an AP model (p<0.001) or an AC model (p<0.001) (Table 1).

**Table 1:** Summary statistics comparing goodness of fit for different COPD mortality maximum likelihood models.

Model	Degree of freedom	Deviance	p Value*
Age	36	4933.8	
Age period (AP)	32	1769.9	<0.001
Age cohort (AC)	24	69.3	<0.001
Age-period-cohort (APC)	21	43.9	

*p Values are based on the Chi square test for comparisons between AP and AC model with the full APC model.

**Figure 3:**
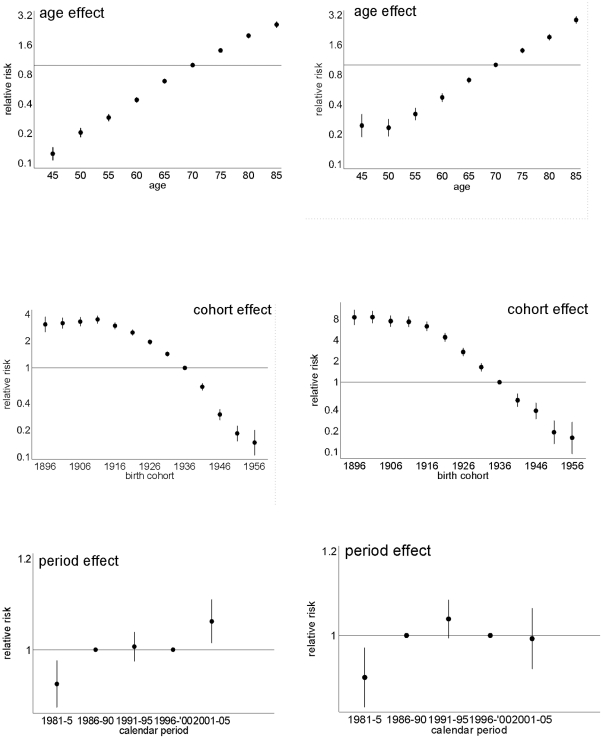
Parameter estimates of age, cohort and period effect from the full APC model for men (left) and women (right).

The age effect (Figure 3) is consistent with the higher COPD mortality observed with age (Figure 1). However, among women but not men, there was also a clear upward inflection around age 50 years. The period effects (Figure 3) have clear downward inflections for both sexes in the calendar year 1991–1995 and possibly an upward inflection 10 years later which was more pronounced for men than women. The cohort effects (Figure 3) have clear downward inflections for men and women around 1911, although overall the decline in the cohort effect from 1911 to 1951 appears to be steeper among women than men. Among men, an initial increase accelerated in birth cohorts from 1896 till 1911, and downturns appeared for the 1930s and 1940s birth cohorts followed by an upturn for the 1950s birth cohorts. Among women there was possibly also a downturn for the 1940s birth cohorts followed by an upturn for the 1950s birth cohorts.

## Discussion

During the period (1981 to 2005) mortality rates for COPD declined in most age groups. However the decomposition by period and cohort reveals a more complex picture, with potential impacts of some macro-level events. The downward inflection in the period curves for both men and women in about 1991 suggests that a common, population-wide exposure decreased vulnerability to COPD mortality at this time, and coincides with the introduction of control on the sulfur content of fuel, which could have both short-term and long-term impacts [8]. Conversely the subsequent upward inflection in the period curve corresponds to a period of increasing air pollution by 10 µg/m^3^ particulates (PM_10_), nitrogen dioxide (NO_2_) and sulphur dioxide (SO_2_) in Hong Kong from 1994 onwards [15]. All of these air pollutants are strongly associated with COPD mortality [16]–[18]. In line with other findings [19], there was a pronounced cohort effect in deaths from COPD. The downward inflections in the cohort curves for both men and women in the 1940s could suggest a decreased generational exposure to smoking, which reduced vulnerability to COPD, although there is no corresponding generational decrease in lung cancer mortality [20]. These downward inflections also coincided with the first generation born and brought up in relatively developed Hong Kong with better childhood living conditions compared with pre-industrial China.

The overall decline in the relative risk of dying from COPD by birth cohort might be due to changes in smoking habits or shortened life time exposure to smoking as quitting has become more common. Smoking prevalence in Hong Kong has been dropping steadily over the years, from 23.3% in 1982 to 11.8% in 2007/8 [9]. Changes in smoking behavior among adults have probably influenced the trends in COPD mortality, as they have also influenced cohort patterns in lung cancer and overall respiratory disease mortality [20]. However, smoking prevalence and smoking amounts in Chinese women are low with fewer than 5% of women in Hong Kong smoking in 2010 [9]. Smoking may not be the only driver of changes in COPD mortality, particularly among women but it has been suggested that women have greater susceptibility than men to the lung-damaging effects of smoking [21].

The downward inflection in 1911 may be an effect of mass migration to Hong Kong in the mid 20^th^ century. The older migrants, pre-1910 birth cohorts, were likely to be healthy survivors who had already exceeded average contemporary Chinese life expectancy [22]–[24].

Overall, the decline in COPD by birth cohort appeared to be greater among women than men. Women in the earlier birth cohorts were mainly migrants from Guangdong Province, where women were traditionally responsible for cooking and may have been more exposed to biomass smoke from cooking than men. Biomass use is associated with COPD [25]–[27]. Given that exposure to biomass smoke from cooking has not been clearly linked with COPD in Hong Kong [28], it is possible that women may have experienced a steeper decline in cumulative lifetime exposure to indoor air pollution than men, hence their greater reduction in COPD mortality by birth cohort than men despite women's low smoking rates throughout.

Exposure to vapors, toxic gas, dust or fumes in the workplaces has been reported to be associated with COPD. A review from the American Thoracic Society estimated the population attributable risk (PAR) for COPD due to occupational exposures ranged up to 37% (median 15%) after adjusting for smoking [29]. Relative to non-smoking, non-occupationally-exposed people, the odds ratio was 1.4 for a never smoker with occupational exposure increasing to 6.2 for people who smoked as well as being exposed to dust and fumes [30]. A higher risk of COPD among men in the 1920s birth cohorts could correspond to greater occupational exposure and increased risk of dying from COPD for those who spent their working lives in industrial occupations in Hong Kong in contrast to later cohorts whose working lives include the current post-industrial era [31]. Moreover, occupational exposures are consistent with deflections for these cohorts being more evident for men than women.

The first generation born and brought up in Hong Kong i.e., the 1940s birth cohorts undoubtedly experienced a step-change in living conditions compared to earlier generations. Starting from 1950, the average annual growth rate of GDP per capita in Hong Kong has been approximately 5% [32]. Also starting in the 1940s, a downturn can be seen in the cohort effect for both men and women. In a wide variety of settings markers of childhood living conditions, such as height or leg length, are consistently negatively associated with respiratory mortality [33]. Growing up in relatively better conditions may promote lung development in childhood via reduced exposure to infections, reduced exposure to smoke from biomass or better nutrition and this would reduce the risk of COPD in later life [34]. Finally, there was a slight upward inflection later in the 1950s; however this is based on very small numbers of deaths and is difficult to explain.

The women smokers appeared to have an increased susceptibility to COPD after the age of 45 to 50 years compared with younger women, which is consistent with a meta-analysis indicating that women are more vulnerable to COPD after the menopause [35]. It is well-known that sex-steroids interact with the immune system, with estrogen enhancing immune function [36], [37]. After the menopause lower estrogen levels may make women more vulnerable to the inflammatory processes involved in COPD. This would be consistent with the observed up turn for this age group.

Despite using population data during a period with well-defined macro-environmental events, the analysis here has a number of limitations. Firstly, the diagnosis of COPD is not always straightforward and this, along with changes in ICD coding, may have added uncertainty to the model. However this is unlikely to have obscured the mortality trends. Moreover, evidence from autopsies suggests that the accuracy of cause of death coding in Hong Kong is similar to that seen elsewhere [38]. Secondly, this is a descriptive study, so we can only speculate about the causes of the changes over time. There are other changes during the period which could have also contributed to the observed changes. The establishment of the Hospital Authority in Hong Kong in the early 1990s may have improved access to care, whereas the Asian financial crisis in the late 1990s may have adversely impacted mortality rates. Thirdly for the younger age groups and the more recent birth cohorts there were relatively few deaths so these points have wide confidence intervals, and should be interpreted with caution. Lastly, COPD is a disease of older people, so we cannot yet determine the life time effects of the high levels of air pollution seen in recent years on the current generation of children. However, the strong cohort effects over generations of better living conditions including less indoor air pollution could be taken as a warning of the potential effects.

From a public health perspective this study provides evidence consistent with modifiable macro-environmental determinants of COPD beyond smoking and passive smoking. Specifically the study suggests detrimental effects of air pollution both indoor and outdoor, but possibly beneficial effects of growing up in a more developed environment. More detailed investigation is required to determine to what extent the gains for COPD prevention from growing up in a more developed environment may be offset by poor outdoor air quality, with corresponding implications for the rest of developing Asia.
